# An All-Solid-State pH Sensor Employing Fluorine-Terminated Polycrystalline Boron-Doped Diamond as a pH-Insensitive Solution-Gate Field-Effect Transistor

**DOI:** 10.3390/s17051040

**Published:** 2017-05-05

**Authors:** Yukihiro Shintani, Mikinori Kobayashi, Hiroshi Kawarada

**Affiliations:** 1 Graduate School of Science and Engineering, Waseda University, 3-4-1 Okubo, Shinjuku, Tokyo 169-8555, Japan; epsilon_koba@asagi.waseda.jp (M.K.); kawarada@waseda.jp (H.K.); 2 R&D Departent, Innovation Center, MK-Hdqrs, Yokogawa Electric Corporation, Japan, 2-9-32 Nakacho, Musashino, Tokyo 180-8750, Japan; 3 The Kagami Memorial Laboratory for Materials Science and Technology, Waseda University, 2-8-26 Nishiwaseda, Shinjuku, Tokyo 169-0051, Japan

**Keywords:** fluorine-termination, pH-insensitivity, polycrystalline boron-doped diamond, electrolyte-solution-gate field-effect transistor, all-solid-state pH sensor

## Abstract

A fluorine-terminated polycrystalline boron-doped diamond surface is successfully employed as a pH-insensitive SGFET (solution-gate field-effect transistor) for an all-solid-state pH sensor. The fluorinated polycrystalline boron-doped diamond (BDD) channel possesses a pH-insensitivity of less than 3mV/pH compared with a pH-sensitive oxygenated channel. With differential FET (field-effect transistor) sensing, a sensitivity of 27 mv/pH was obtained in the pH range of 2–10; therefore, it demonstrated excellent performance for an all-solid-state pH sensor with a pH-sensitive oxygen-terminated polycrystalline BDD SGFET and a platinum quasi-reference electrode, respectively.

## 1. Introduction

After Bergveld [1] first presented an ion-sensitive field-effect transistor (ISFET), various types of ISFET have been presented, i.e., silicon-based ISFETs (Si-ISFETs) with an insulator of tantalum pentaoxide (Ta_2_O_5_), with silicon nitride (Si_3_N_4_), with aluminum oxide (Al_2_O_3_) [2], and with a diamond-like carbon insulator [3]. We recently reported no-gate-insulator electrolyte-solution-gate field-effect transistors (SGFETs) with a single crystal diamond surface channel [4,5,6,7], with a polycrystalline diamond surface channel, and with a boron-doped diamond surface channel [8,9,10,11]. The ISFET has been a promising candidate for an integrated device to realize a high-response chemical sensor at a low cost. However, ISFET sensors have not been widely used in a practical way, in spite of the advantage of its feasibility with regard to miniaturization. One of the reasons is that a miniaturized ISFET requires a miniaturized stable reference system, which is still a challenging research topic. A silver/silver chloride (Ag/AgCl) gate electrode is commonly applied for pH sensing, but it is a liquid-containing electrode. To realize an all-solid-state sensor, two categories of on-chip reference systems have been studied. One of the approaches is the on-chip fabrication of an Ag/AgCl gate electrode with a gel filled in a hole [12,13], but the leakage of the gel resulted in the contamination of the sample, or in a limited lifetime. Another approach is to use a differential FET sensing technique, which is a combination of an ISFET and a pH-insensitive FET (REFET: reference field-effect transistor). The first REFET introduced by Matsuo [14] was an identical ISFET that possessed a chemically insensitive layer. Janata’s REFET used an ISFET and a quasi-reference electrode (QRE) with the differential FET sensing technique [14]. Various types of REFETs were reported including buffered hydrogels, silanes, polyacetal, and parylene [15]. However, the realization of a REFET, which possesses enough pH-insensitivity compared with ISFETs with typically 30–60 mV/pH sensitivity, has been a struggle for the differential FET sensing technique that results in an all-solid-state pH sensor. In the REFET/ISFET arrangement, a metal is used for quasi-reference electrode (QRE), placed close to the REFET/ISFET, which formally replaces the conventional reference electrode. In this case, the interface between the QRE and the electrolyte may be thermodynamically uncontrolled, and thus may generate an unstable voltage. Nevertheless, those voltages can be obtained by the REFET/ISFET differential sets as a common voltage mode that does not influence the sensor output voltage of the differential sensing technique. A pH change in the solution is measured by the differential output voltage as the ISFET responds with high sensitivity and the REFET responds with less sensitivity. Various types of REFETs are reported for differential FET sensing, such as a hydrogel as an insensitive membrane and a parylene layer for an ion blocking material [12]. Another approach is to use specific-ion-sensitive polymeric membranes such as poly (vinyl chloride) (PVC), whose pH sensitivity for protons is lower than for other cations. However, PVC is considered not suitable for REFET as it typically shows cation permselectivity. This behavior is a common property of polymeric membranes, meaning that polymer membranes are unsuitable for the REFET layer [12].

Here, in order to overcome the abovementioned problems, we first proposed introducing a fluorine-terminated diamond for a pH-insensitive SGFET, which worked in conjunction with an oxygen-terminated diamond channel employing a pH-sensitive SGFET.

## 2. Experimental

### 2.1. SGFET Fabrication

A boron-doped polycrystalline diamond SGFET was fabricated by the protocol described in our previous reports [9,10]. To fabricate a boron-doped layer, polycrystalline diamond substrates of 10-mm square, purchased from Materials and Electronics Co. Ltd., were used throughout. Boron-doping diamond layers were deposited using a quartz-type microwave chemical vapor deposition (CVD) reactor. The typical CVD conditions were identical to our previous research [8,9]. Two titanium/gold (typically 20 nm/100 nm) contact pads were deposited for drain and source electrodes on the as-grown hydrogen-terminated BDD surface, and then they were patterned with lift-off photolithography techniques with the help of the same process used in our previous work [8,9]. The contact metal pads were positioned to give a channel length of 5–10 mm and a channel width of 0.3–0.6 mm, and electric wires were connected to the drain and source titanium/gold pads with a conductive adhesive for applying external bias voltage. Then, the drain and source electrodes were encapsulated with nonconductive epoxy resin to protect them from the solution; only the area of the boron-doped diamond surface between the pads was exposed to the buffer solutions.

### 2.2. Surface Modification of Diamond for pH-Insensitive/pH-Sensitive BDD SGFET

**Figure 1:**
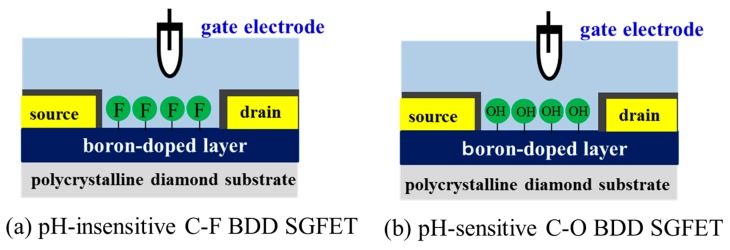
Schematic of a pH-insensitive fluorine-terminated boron-doped diamond (BDD) solution-gate field-effect transistor (SGFET). (**a**) pH-insensitive fluorine-terminated BDD (C-F BDD) SGFET; (**b**) pH-sensitive oxygen-terminated BDD (C-O BDD) SGFET.

To decrease/improve the pH sensitivity of BDD SGFET sensor, the direct-wetted diamond surface, employed as the FET channel, was modified to partially fluorine-terminated BDD and partially oxygen-terminated BDD with the help of the method used in the previous study [8,9,10,11,16]. Figure 1a,b illustrate the schematic diagrams of the pH-insensitive fluorine-terminated BDD (C-F BDD) SGFET surface and the pH-sensitive oxygen-terminated BDD (C-O BDD) SGFET surface. The transformation coverage percentage of the C-F group and C-O group from the as-grown C–H diamond surface depends of the surface treatment method and condition. The surface treatment of partial fluorination was conducted using inductively coupled plasma (ICP) with an octafluoropropane (C_3_F_8_) gas source at a pressure of 2–5 Pa at 300–600 W for 10–120 s. To improve the pH sensitivity for SGFET, partial oxidation of the BDD surface was conducted by ultraviolet irradiation in a chamber with an oxygen atmosphere, a method that was used in our previous work [8,9]. For the estimation of the coverage of the C-F functional group of the surface, the BDD channel after fluoridation treatment was analyzed by using a ULVAC-Phi Model 3300 X-ray photoelectron spectrometer.

### 2.3. Measuring System for Characterization of FET

An individual FET measurement was characterized by using the measurement system comprising Keithley Instruments Model 2400 source-measure units (Keithley Instruments, Cleveland, OH, USA) and YOKOGAWA Model GS820 source measure units (Yokogawa, Tokyo, Japan) for the application of drain-source voltage and gate-source voltage with a common-source mode. Data acquisition with processing was performed using Labview 2010 software (National Instruments, Austin, TX, USA) to maintain a constant source-drain current by applying a compensating gate-source voltage. The gate-source voltage was applied via an Ag/AgCl gate electrode for characterization of the pH sensitivity. All BDD SGFET I-V profiles were obtained at room temperature. For the characterization of differential FET pH sensing, a lab-made ISFET pH-mV meter, equipped with a source-follow circuit suitable for the BDD SGFET with P-channel semi-conductivity, was used to measure the FET source-gate voltage corresponding to the solution pH and the differential output of FETs. Three types of buffer solution were used for evaluation: a 1 millimolar PBS (phosphate-buffered saline) buffer solution (pH 7.4), four kinds of pH standard solutions of 4.01 (phthalate buffer), 6.86 (phosphate buffer), 9.18 (tetraborate buffer), and 10.01 (carbonate buffer), and a wide-range buffer solution (so-called Carmody pH buffer) whose pH range was adjusted from 2 to 12 by mixing 0.1 molar trisodium phosphate (dodecahydrate), 0.2 molar boric acid, and 0.05 molar citric acid (monohydrate). All chemicals used in this study were of reagent-grade and were obtained from Tokyo Kasei, Horiba, Kanto Kagaku, and Wako.

## 3. Results and Discussion

### 3.1. Characterization of pH-Insensitive C-F BDD SGFET

**Figure 2:**
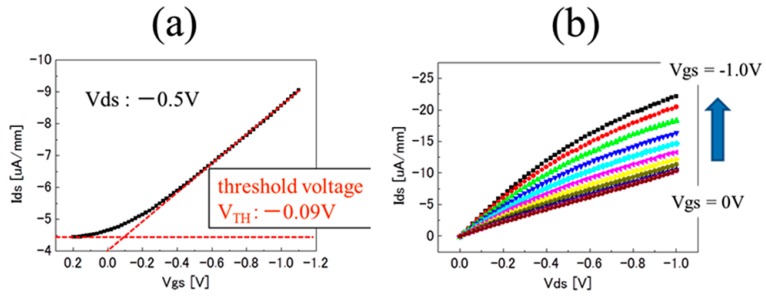
Device properties of an electrolyte-solution-gate diamond FET (SGFET) with a fluorine-terminated boron-doped layer in a 1 molar PBS buffer solution (pH 7.4). (**a**) Ids-Vgs characteristics at Vds = −0.5 V in the range of −1.0 V to 0.2 V; (**b**) Ids-Vds characteristics at Vgs in the range of −1.0 V to 0 V.

The specification of the boron-doped diamond film of the BDD SGFET used in this study was the same as our previous study [8]. By AC Hall measurements, the sheet carrier density, mobility, and sheet resistance of the BDD film before oxygen treatment was 3.7 *×* 10^13^/cm^2^, 8.6 cm^2^/Vs, and 24 kΩ/cm^2^, respectively. The FET characteristics of the C-F BDD SGFET were examined. For the estimation of the coverage of the C-F functional group of the surface, the BDD channel after fluoridation treatment was analyzed by X-ray photoelectron spectrometer. In the case of C_3_F_8_ ICP plasma condition of 100 W for 30 s, the peak height calculation of F1s/(C1s + F1s) was 0.30. In contrast, the peak height of F1s of the BDD-SGFET surface without fluorination treatment was below the limit of quantification. For the characterization of the fluorine-terminated polycrystalline boron-doped diamond as a pH-insensitive SGFET, a fluorine-terminated BDD (C-F BDD) SGFET and an Ag/AgCl gate electrode were immersed in a 1 molar PBS buffer solution (pH 7.4) for 10 min for initialization, and then its FET current–voltage (FET I-V) characteristics were evaluated by the common-source method in two ways: via the drain-source current (Ids)-drain-source voltage (Vds) characteristics and via the drain-source current (Ids)-gate-source (Vgs) characteristics (the so-called static characteristics). Figure 2a shows the Ids-Vgs characteristics of a C-F BDD SGFET, as evaluated using the electrolyte-gate-bias voltage mode with the Vgs varying from −1.0 V to 0.2 V at Vds −0.5 V. The Ids-Vgs characteristics exhibit a linear relationship in the Vgs range from ca. −0.3 V to greater than −1.0 V, which is beneficial for the SGFET sensor. On the basis of the gate-bias intercept of the extrapolation of the drain-source current curve in the transfer characteristics (dash-dash line), the threshold voltage of ca. −0.09 V was estimated in the normally-on mode. The static characteristics (Ids-Vds characteristics) of the C-F BDD SGFET are shown in Figure 2b. The Vgs applied through an Ag/AgCl gate electrode was varied stepwise from −1.0 V to 0 V in steps of 0.1 V. For each value of Vgs, the Ids was obtained as a function of the Vds from −1.0 V to 0 V. The drain-source current was pinched off, and the distinct linear and saturation regions were obtained in the Ids-Vds profiles.

### 3.2. pH-Insensitivity of C-F BDD SGFET and pH-Sensitive C-O BDD SGFET

**Figure 3:**
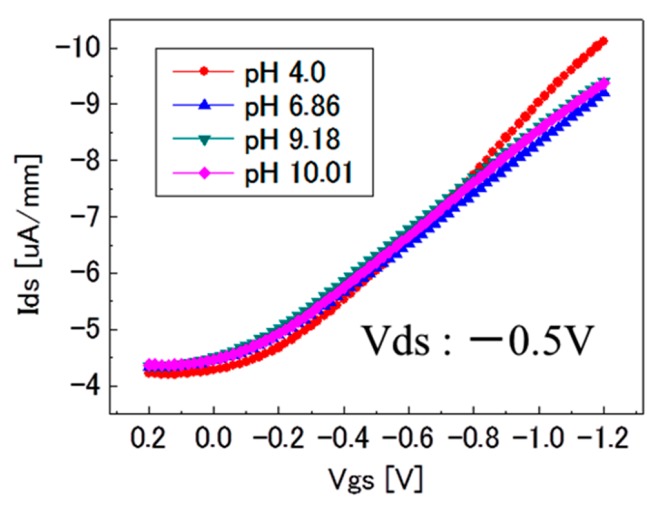
Vgs-Ids curve of pH-insensitive C-F BDD SGFET with the condition of Vds = 0.5 V and Ids = −0.7 µA/mm.

**Figure 4:**
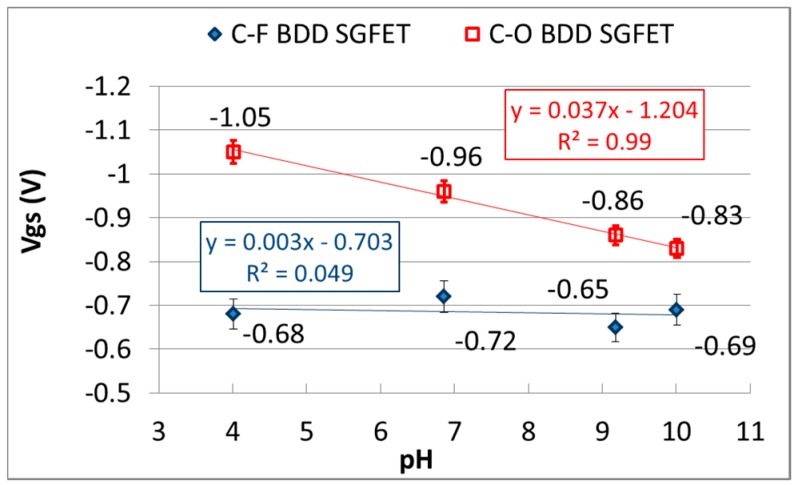
pH-insensitivity of the C-F BDD SGFET and pH-sensitivity of the C-O BDD SGFET.

For the evaluation of the pH-insensitivity of C-F BDD SGFET and the pH-sensitivity of oxygen-terminated BDD (C-O BDD) SGFET, the experimental results of the FET-IV profiles were obtained using pH standard solutions such as pH = 4.01 (phthalate buffer), 6.86 (phosphate buffer), 9.18 (tetraborate buffer) and 10.01 (carbonate buffer) with reference to JIS (Japanese Industrial Standard) K0802 and JIS Z 8802. Figure 3 shows the FET-IV characteristics of pH-insensitive C-F BDD SGFET. A family of Ids-Vgs curves were generated by applying a series of step voltages at the Ag/AgCl gate electrode. The pH-insensitivity of the C-F BDD SGFET at fixed Ids and Vds values was calculated from the data shown in Figure 3. The obtained results are shown in Figure 4 as a blue box plot with an error bar that indicates the standard deviation (*n* = 5). The sensitivity was less than ca. 3 mV/pH at Ids = −7 µA/mm. The Vgs decreased with the pH over the entire investigated pH range of pH 4.01 to pH 10.01. By contrast, the pH-sensitive C-O BDD SGFET was also evaluated. The C-O BDD SGFET used in this work was prepared with the help of the same process described in our previous study [9]. The sensitivity of the C-O BDD SGFET is also shown in Figure 4 as a red rhombus point. The sensitivity is 37 mV/pH, which is at least 10 times higher than that of the sensitivity of the C-F BDD SGFET and is enough to be employed as a pH-sensitive SGFET.

### 3.3. Differential FET Sensing, Using pH-Insensitive C-F BDD SGFET and pH-Sensitive C-O BDD SGFET

**Figure 5:**
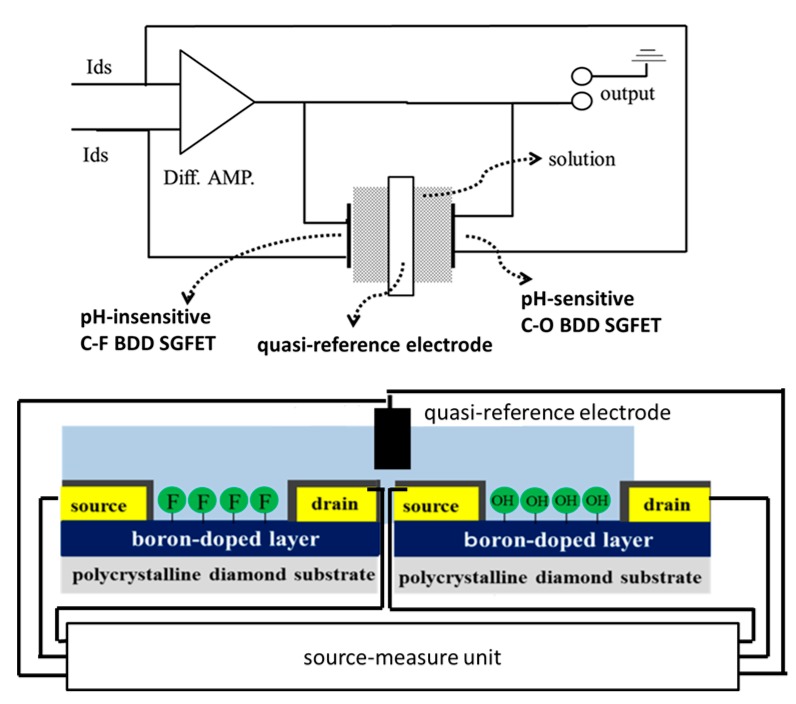
Schematic diagram of differential FET measurement using the source-follower circuit.

**Figure 6:**
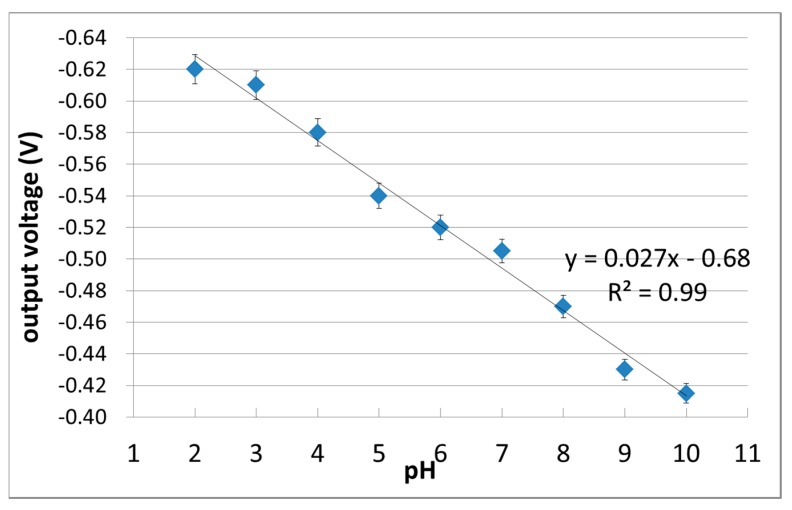
pH sensing of the differential FET. The differential output voltage of the pH-insensitive C-F BDD SGFET and the pH-sensitive C-O BDD SGFET are plotted.

A device specification of the differential FET using pH-insensitive C-F BDD SGFET and C-O BDD SGFET was examined by a lab-made ISFET pH-mV meter equipped with a source-follow circuit. Figure 5 is a schematic diagram of the differential FET measurement using the source-follow circuit. Figure 6 shows the experimental results of the all-solid-state pH sensor with a pH-sensitive C-O BDD SGFET and a pH-insensitive C-F BDD SGFET, with an error bar that indicates the standard deviation (*n* = 4). The horizontal axis is the pH of the solution, and the vertical axis is the differential output voltage. The output was shifted with linearity corresponding to the pH. A good output response was obtained with good linearity between pH 2 to 10 with R^2^ = 0.99. These results suggest that the combination of the pH-insensitive C-F BDD SGFET, the pH-sensitive C-O BDD SGFET, and platinum QRE was employed as the all-solid-state pH sensor.

The short-term stability of the all-solid-state pH sensor was examined with reference to JIS (Japanese Industrial Standard) K0802 and JIS Z 8802. The 24 h stability test using five sensors were delta-pH of 0.096 with variation coefficient CV = 25%, where the JIS standard of delta-pH is below 0.1.

## 4. Conclusions

In this study, a method of fabricating a pH-insensitive BDD SGFET by introducing a C-F diamond surface was proposed. With differential FET sensing, a sensitivity of 27 mv/pH was obtained in the pH range of 2–10; thus, it demonstrated excellent performance of an all-solid-state pH sensor with the pH-sensitive C-O BDD SGFET and platinum employed as the pH-sensitive SGFET and the QRE, respectively. The short-time and long-time drift characteristics and the interference of other ions/chemicals will be examined from a practical application perspective.

## Abbreviations

ISFET	ion-sensitive field-effect transistor
SGFET	solution-gate field-effect transistor
REFET	reference field-effect transistor
C-O BDD	oxygen-terminated boron-doped diamond
C-F BDD	fluorine-terminated boron-doped diamond
QRE	quasi-reference electrode
BDD	boron-doped diamond
Ids	drain-source current
Vgs	gate-source voltage
Vds	drain-source voltage
PVC	poly (vinyl chloride)
ICP	inductively coupled plasma
CVD	chemical vapor deposition
